# Successful removal of a migrated biliary plastic stent using a novel spiral dilator

**DOI:** 10.1055/a-2098-0982

**Published:** 2023-06-15

**Authors:** Yusuke Ishida, Takehiko Koga, Naoaki Tsuchiya, Kaori Hata, Kei Nishioka, Noriko Shiga, Fumihito Hirai

**Affiliations:** 1 Department of Gastroenterology and Medicine, Fukuoka University, Faculty of Medicine, Fukuoka, Japan; 2 Department of Gastroenterology, Fukuokaken Saiseikai Futsukaichi Hospital, Fukuoka, Japan

Endoscopic plastic stenting is an established biliary drainage technique. However, plastic stents may migrate proximally or distally, causing recurrent biliary obstruction 1 . The Tornus ES (Olympus Co., Tokyo, Japan) is a newly designed coil-sheath dilator with a screw-shaped tapered tip ( Fig. 1 ) that has been recently reported in endoscopic interventions 2 3 4 5 . We herein introduce successful troubleshooting using this novel spiral dilator for removal of a migrated biliary plastic stent.

**Fig. 1:**
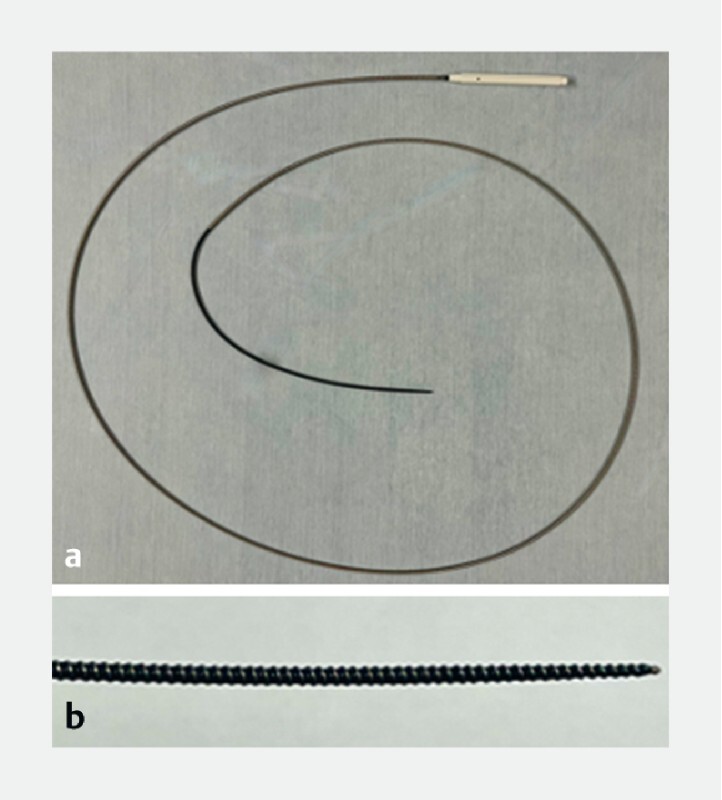
Novel spiral dilator (Tornus ES). **a** The dilator is composed of a coil sheath and handle to rotate. The maximal outer diameter is 7 Fr. **b** The tip of the dilator is screw-shaped and tapered, allowing it to advance over the guidewire. There are two lines dedicated to 0.025- and 0.018-inch guidewires.

A 63-year-old man presented with obstructive jaundice caused by pancreatic head cancer. Because he had undergone biliary drainage using an 8.5-Fr plastic stent with sphincterotomy 2 months before, we attempted to exchange the stent. The tumor had invaded the duodenum, and the plastic stent had almost migrated ( Fig. 2 ). We chose to use a novel spiral dilator (Tornus ES) because a 0.025-inch guidewire could be introduced into the stent. Its tip was inserted into the distal end of the stent over the guidewire, and care was taken not to push the stent up ( Fig. 3 a ). It was then advanced smoothly into the inside of the stent with clockwise rotation. Once the stent and spiral dilator were engaged, we confirmed that the stent rotated in accordance with the movement of the spiral dilator under endoscopic or fluoroscopic vision ( Fig. 3 b ). Finally, the stent was successfully removed with the spiral dilator through the scope channel ( Fig. 3 c , Video 1 ).

**Fig. 2:**
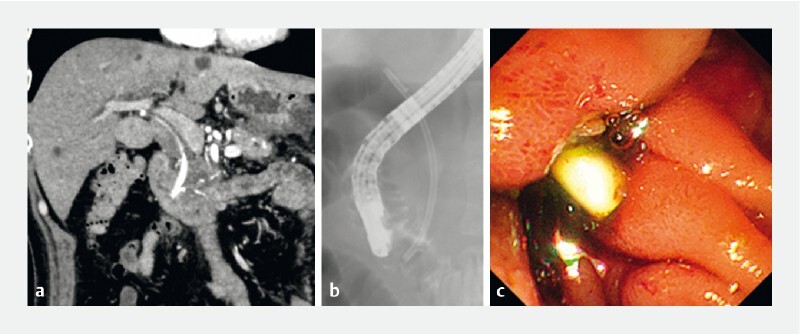
A migrated biliary stent. **a** A computed tomography image showing pancreatic cancer and a plastic stent. Endoscopic biliary drainage had been performed using an 8.5-Fr plastic stent. The pancreatic cancer had invaded not only the distal bile duct but also the duodenum. **b** Fluoroscopic image revealed duodenal stricture around the periampullary area without gastric output obstruction. **c** Endoscopic image of the plastic stent, which had almost migrated into the bile duct.

**Fig. 3:**
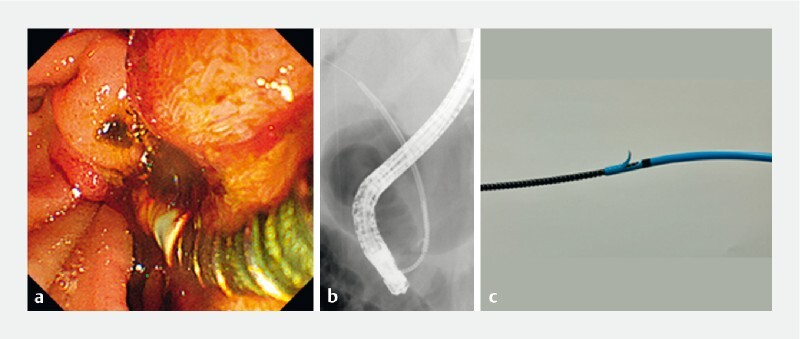
Stent removal using the spiral dilator. **a** Endoscopic image showing that the spiral dilator was inserted into the distal end of the stent. **b** Fluoroscopic image showing that the spiral dilator was inserted into the distal end of the stent through the guidewire. **c** Ex vivo image of the spiral dilator and the same stent as the migrated stent. The spiral dilator and 8.5-Fr plastic stent are engaged.

**Video 1** Endoscopic removal of a migrated biliary plastic stent using a novel spiral dilator.


Although the outer diameter of the Tornus ES is 7 Fr, it could be inserted and engaged with 7-, 8.5-, and 10-Fr plastic stents in an ex vivo trial ( Fig. 4 b, c, d ). In contrast, the tapered tip of the Tornus ES could not be inserted into a 6-Fr stent ( Fig. 4 a ). Depending on the inner diameter of the stent, there is a high possibility that plastic stents of 7-Fr or larger can be removed. This case demonstrates that removal of a migrated plastic stent using Tornus ES can be a troubleshooting option.

**Fig. 4:**
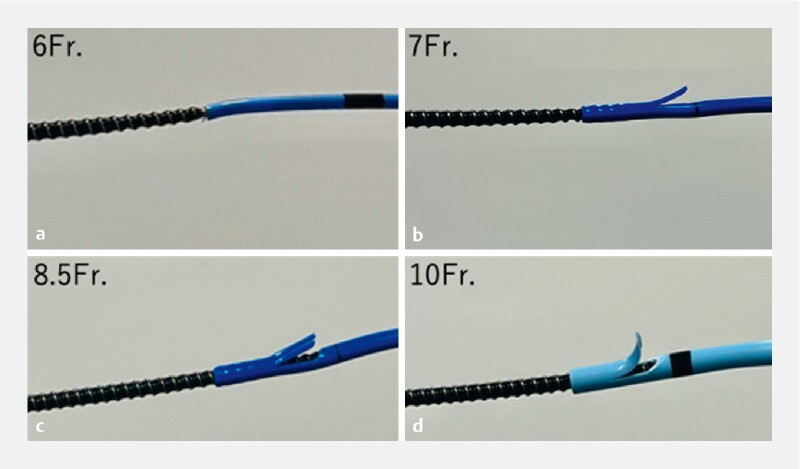
Ex vivo trial. **a** The spiral dilator cannot be inserted into and engaged with a 6-Fr stent. **b, c, d** The spiral dilator can be inserted and engaged with plastic stents of 7 Fr or larger.

Endoscopy_UCTN_Code_TTT_1AR_2AZ

## Notes

Endoscopy E-Videos

https://eref.thieme.de/e-videos
*E-Videos* is an open access online section of the journal *Endoscopy* , reporting on interesting cases and new techniques in gastroenterological endoscopy. All papers include a high-quality video and are published with a Creative Commons CC-BY license. Endoscopy E-Videos qualify for HINARI discounts and waivers and eligibility is automatically checked during the submission process. We grant 100% waivers to articles whose corresponding authors are based in Group A countries and 50% waivers to those who are based in Group B countries as classified by Research4Life (see: https://www.research4life.org/access/eligibility/ ). This section has its own submission website at https://mc.manuscriptcentral.com/e-videos

## References

[1] Okabe Y Tsuruta Y Kaji Y Endoscopic retrieval of migrated plastic stent into bile duct or pancreatic pseudocyst Dig Endosc 2009 21 1 7 19691793 10.1111/j.1443-1661.2008.00818.x

[2] Yasuda T Hara K Haba S Dilation of pancreatic duct stenosis using a newly designed drill dilator Dig Endosc 2022 34 e73 e74 35318735 10.1111/den.14269

[3] Mandai K Inoue N Removal of biliary plastic stent using a novel spiral dilator for reliable stent placement Dig Endosc 2022 34 e168 e169 36250289 10.1111/den.14439

[4] Yamada M Hara K Haba S Endoscopic ultrasound-guided hepaticogastrostomy using a novel drill dilator Endoscopy 2022 54 E856 E857 35636451 10.1055/a-1838-3682 PMC9735340

[5] Yamada M Okamoto T Sasahira N Troubleshooting with a drill dilator for the stent-in-stent technique in malignant hilar biliary obstruction Endoscopy 2023 55 E189 E190 36368673 10.1055/a-1956-1266 PMC9829803

